# Chitosan oligosaccharides packaged into rat adipose mesenchymal stem cells-derived extracellular vesicles facilitating cartilage injury repair and alleviating osteoarthritis

**DOI:** 10.1186/s12951-021-01086-x

**Published:** 2021-10-26

**Authors:** Shenglong Li, Jie Liu, Siyu Liu, Weijie Jiao, Xiaohong Wang

**Affiliations:** 1 Department of Tissue Engineering, Center of 3D Printing & Organ Manufacturing, School of Intelligent Medicine, China Medical University (CMU), No. 77 Puhe Road, Shenyang North New Area, Shenyang, 110122 China; 2 Department of Bone and Soft Tissue Tumor Surgery, Cancer Hospital of China Medical University, Liaoning Cancer Hospital & Institute, Shenyang, 110042 Liaoning China; 3 Department of Prosthodontics, School and Hospital of Stomatology, China Medical University, Liaoning Provincial Key Laboratory of Oral Diseases, Shenyang, 110002 China; 4 Center of Organ Manufacturing, Department of Mechanical Engineering, Tsinghua University, Beijing, 100084 China

**Keywords:** EVs, Chitosan oligosaccharides, EVs-COS conjugates, Cartilage injury repair, Osteoarthritis

## Abstract

**Objectives:**

This study aimed to investigate the roles of adipose mesenchymal stem cell (AMSC)-derived extracellular vesicles (EVs) binding with chitosan oligosaccharides (COS) in cartilage injury, as well as the related mechanisms.

**Results:**

IL-1β treatment significantly inhibited the viability and migration of chondrocytes and enhanced cell apoptosis (*P* < 0.05), while chitosan oligosaccharides and extracellular vesicles-chitosan oligosaccharide conjugates (EVs-COS/EVs-COS conjugates) reversed the changes induced by IL-1β (*P* < 0.05), and the effects of extracellular vesicles-chitosan oligosaccharide conjugates were better than those of chitosan oligosaccharides (*P* < 0.05). After cartilage damage, IL-1β, OPN, and p53 were significantly upregulated, COL1A1, COL2A1, OCN, RUNX2, p-Akt/Akt, PI3K, c-Myc, and Bcl2 were markedly downregulated, and extracellular vesicles-chitosan oligosaccharide conjugates reversed the expression induced by cartilage injury. Through sequencing, 760 differentially expressed genes (DEGs) clustered into four expression patterns were associated with negative regulation of the canonical Wnt, PI3K-Akt, AMPK, and MAPK signaling pathways.

**Conclusion:**

Extracellular vesicles-chitosan oligosaccharide conjugates may serve as a new cell-free biomaterial to facilitate cartilage injury repair and improve osteoarthritis.

**Graphical Abstract:**

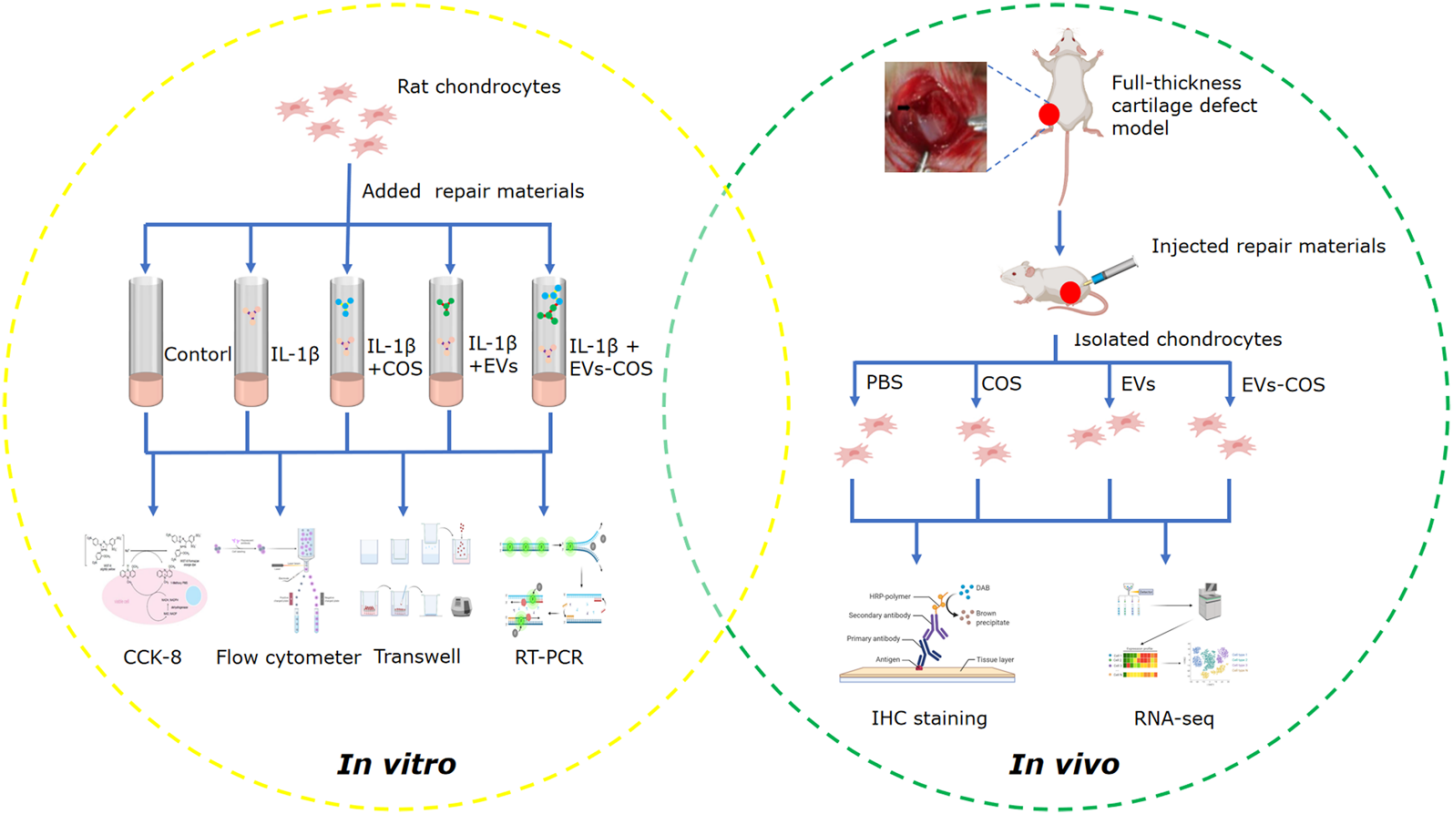

**Supplementary Information:**

The online version contains supplementary material available at 10.1186/s12951-021-01086-x.

## Background

Articular cartilage, a non-self-repairing tissue, is mainly composed of water, proteoglycan, and collagen, which together determine the functional characteristics of cartilage tissues [1]. Cartilage injury usually marks the occurrence of tissue degeneration, progressive deterioration, subchondral osteosclerosis, and osteoarthritis (OA) [2]. OA clinically manifests as slow progression of joint pain, tenderness, stiffness, joint swelling, limited movement, and joint deformity [3], and is a major cause of disability, affecting approximately 240 million people globally [4]. Currently, drugs used to alleviate the symptoms of OA include steroid injections, non-steroidal anti-inflammatory drugs (NSAIDs), and opioids [5, 6]. However, the long-term use of these drugs may result in side effects, such as gastrointestinal, renal, and cardiovascular diseases [7]. Nanocomposites, including organic–inorganic, inorganic-inorganic, and bioinorganic nanomaterials, have been reported in bone tissue regeneration engineering, such as hydroxyapatite (HA) with chitosan, polycaprolactone/bioglass, and HA-gelatin nanocomposites [8]. By combining nanotechnology-based drug delivery systems, the bioavailability, pharmacokinetics and pharmacodynamics of drugs in bone tissue can be improved, thus improving therapeutic efficacy while reducing side effects. Owing to the immunogenicity of the receptor cells, rapid blood clearance, cytotoxicity, and poor biological distribution of these nanocomposites, their use has been limited [9]. Therefore, more therapeutic strategies are urgently needed to improve cartilage injury repair and manage OA.

Mesenchymal stem cells (MSCs), which can be isolated from many adult organs, are self-renewing multipotent progenitors, and can differentiate into a variety of cell lineages, such as adipocytes, osteoblasts, and chondrocytes [10]. Increasing evidence has shown that MSC transplantation promotes tissue regeneration, including fracture, wound healing, and cartilage repair [11]. A previous study indicated that bone marrow MSCs could enhance articular cartilage repair and regeneration, as well as improve the quality of life of knee OA [12]. In addition, owing to their relatively easy isolation, high yield, and strong potential for proliferation and differentiation [13], adipose MSCs (AMSCs) have been widely used in various biomedical applications. A previous double-blinded clinical trial showed that intraarticular injection of AMSCs could improve cartilage defects and relieve pain in knee OA patients, without causing adverse events at 6 months' follow-up [14]. Another study reported that AMSCs with BMP9 overexpression promoted cartilage repair and differentiation through the Notch1/Jagged1 signaling pathway [15]. These findings suggest that AMSCs can promote cartilage injury repair and improve OA. Nevertheless, the clinical effects of traditional AMSC transplantation methods have been greatly limited by their stability, safety, and immune-mediated rejection.

Extracellular vesicles (EVs) are released by a variety of cells [16], and can serve as a tool for cell-to-cell communication. They can selectively encapsulate protein molecules, genes (RNA and DNA), cytokines, and other functional bioactive substances derived from the cells and deliver them to the extracellular environment or other target cells [17]. EVs derived from AMSCs have been considered an important part of cell-free regenerative medicine because they carry special bioactive substances and possess the distinctive and typical characteristics of AMSCs [18]. Many studies have confirmed that AMSC-derived EVs play essential roles in skin healing and regeneration, neurodegenerative diseases, ischemia reperfusion injury, and obesity [19]. A recent study demonstrated that AMSC-derived exosomal microRNA (miR)-19b could enhance the ability to heal wounds by targeting the CCL1/TGF-β signaling axis [20]. Another study also reported that AMSC-derived EVs promoted cartilage formation by upregulating miR-145 and miR-221, and inhibited inflammation by downregulating pro-inflammatory markers (IL-6, NF-κB, and TNF-α) [21]. In addition, EVs have better targeting ability and safety and are attracting increasing attention. Therefore, AMSC-derived EVs are expected to be a new clinical strategy for cartilage injury repair and OA treatment.

Chitosan, a natural source of polysaccharides, has been widely used in biomedical fields, such as tissue engineering and pharmaceutical preparation, owing to its biodegradable, biocompatible, and non-toxic properties [22]. Previous studies have shown that chitosan has antibacterial and anticoagulant properties, can promote wound healing, and can improve immune functions [23–25]. Carboxymethyl chitosan combined with hydroxyapatite has been reported to enhance alkaline phosphatase (ALP) activity and promote osteoblast differentiation and new bone formation/maturation in vivo and in vitro, and may therefore, be used as a promising scaffold for bone tissue engineering [26]. Chitosan/poly (vinyl alcohol) could allow AMSCs to proliferate and differentiate into chondrocytes, improving their clinical effects on OA pathology. A previous study used AMSC-derived exosomes to immobilize the polydopamine-coated PLGA (PLGA/pDA) scaffolds, and found that exosomes could be released from PLGA/pDA scaffolds and significantly promote bone formation and regeneration of bone tissue [27]. Therefore, we speculate that chitosan can be used as a scaffold to promote the effects of AMSC-derived EVs. However, the roles of chitosan combined with AMSC-derived EVs in cartilage injury repair and the underlying mechanisms remain unclear.

In this study, both in vitro and in vivo rat cartilage injury models were constructed to systematically investigate the effects and related mechanisms of low molecular chitosan oligosaccharides (COS) packaged into rat AMSC-derived EVs in cartilage injury. These findings provide new insights and strategies for drug development for the treatment of OA and other degenerative joint diseases based on cartilage injury.

## Results

### Determination of the optimal concentrations of EVs and COS

To determine the optimal concentrations of EVs and COS for further experiments, chondrocytes were treated with different concentrations of EVs and COS for 48 h, after which their viability was determined. As shown in Additional file 1: Figure S1 A, cell viability after exposure to 5, 10, 20, and 50 μg/mL EV treatment was significantly increased compared with the untreated cells (*P* < 0.05), and the cell viability was the highest in the cells treated with 20 μg/mL EVs (Additional file 1: Figure S1A). Furthermore, there was no significant difference in cell viability among the cells treated with 0, 10, and 20 μg/mL COS (*P* > 0.05, Additional file 1: Figure S1B). When the cells were treated with 40, 80, 160, 320, 640, and 1280 μg/mL COS, their viability was significantly enhanced (*P* < 0.05), and the viability of the cells treated with 320 μg/mL COS was the highest (Additional file 1: Figure S1B). Based on these results, 20 μg/mL EVs and 320 μg/mL COS were selected for subsequent experiments.

### Identification of rat AMSCs-derived EVs, EVs-COS, and rat chondrocytes

**Fig. 1:**
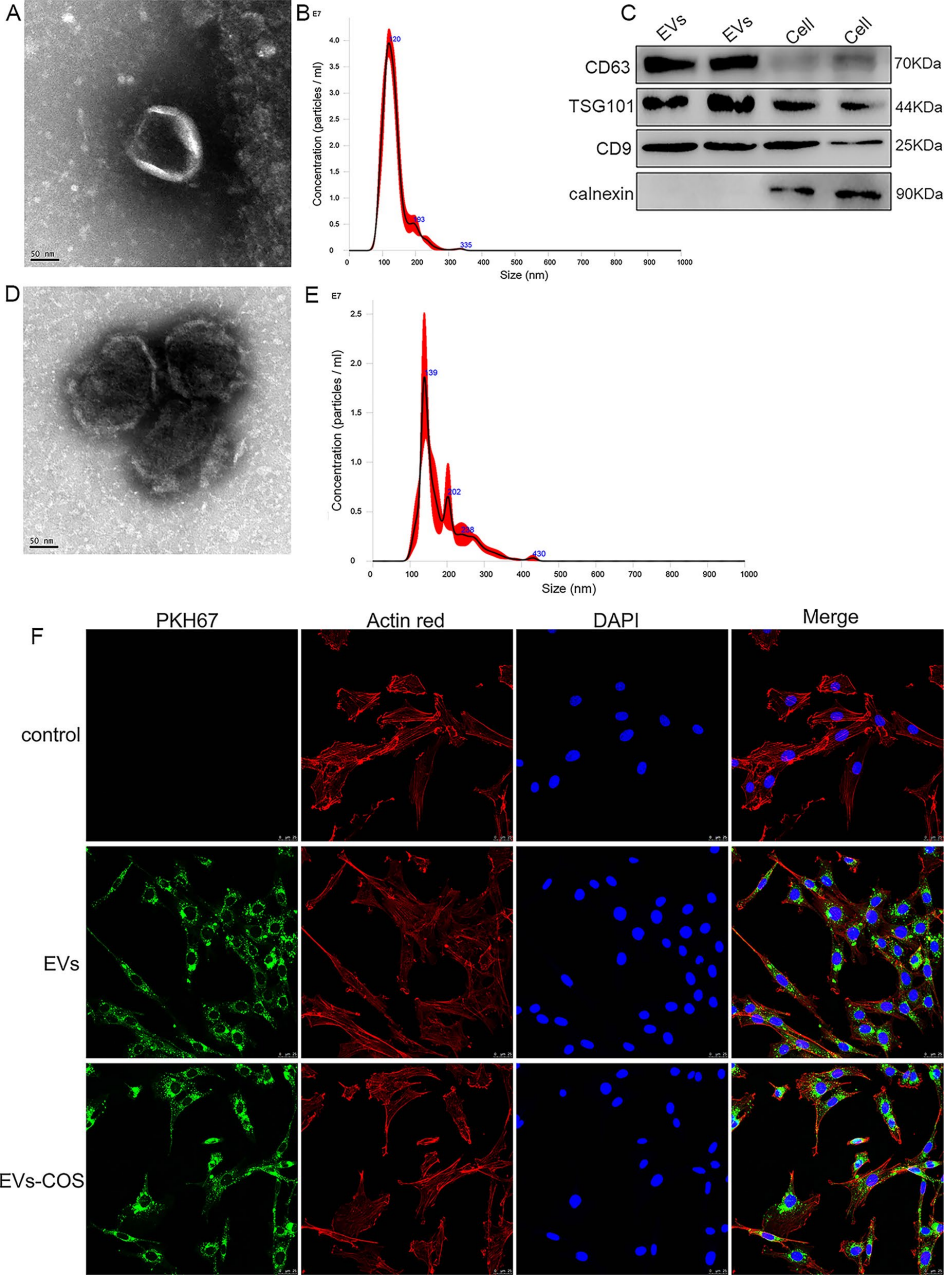
Characterization of extracellular vesicles (EVs) isolated from rat adipose mesenchymal stem cells (AMSCs), and EVs-chitosan oligosaccharide (COS) conjugates (EVs-COS). **A** The morphology of rat AMSCs-derived EVs observed by transmission electron microscopy (TEM). **B** The particle size distribution of EVs determined by a Nanosight NS300 particle size analyzer (NTA). **C** The expressions of EVs surface markers (CD63, TSG101, and CD9) measured using Western blot. **D** The morphology of EVs-COS by using TEM. **E** The particle size distribution of EVs-COS measured by NTA. **F** EVs and EVs-COS taken up by rat chondrocytes visualized using PKH67 staining kit (green fluorescence)

EVs isolated from rat AMSCs were characterized using TEM, NTA, and Western blotting. TEM showed that the extracted substances were cup-shaped or nearly round with a diameter of approximately 100 nm (Fig. 1A). The results of NTA showed that the major peak of isolated substances was approximately 120 nm, and that the average peak was 133.9 ± 0.8 nm (Fig. 1B), which was in accordance with the previously reported size distribution of EVs [28]. In addition, Western blot results demonstrated that CD3, TSG101, and CD9, which are EV markers, were all expressed in the EVs, whereas calnexin was only expressed in the cells (Fig. 1C). These results indicate that EVs were successfully isolated from rat AMSCs.

After COS bound with EVs, TEM and NTA were used to observe the morphology and determine the size of the extracellular vesicles-chitosan oligosaccharide conjugates (EVs-COS), respectively. The morphology of EVs-COS was nearly round or round with a diameter of approximately 100 nm (Fig. 1D), which is consistent with the characteristics of EVs. NTA results showed that the major peak of EVs-COS was 139 nm, and that the average peak was 183.4 ± 1.5 nm (Fig. 1E), which indicated that the size of EVs-COS was higher than that of EVs. The TEM and NTA results confirmed that COS successfully bound to EVs.

In addition, chondrocytes isolated from the cartilage tissues of rats were identified by determining the expression of collagen II. Additional file 2: Figure S2 shows that the cells were stained dark yellow–brown, a typical characteristic of isolated chondrocytes. Positive results were denoted by the staining of the collagen II components.

### EVs and EVs-COS uptake by chondrocytes

PKH67 was used to label EVs and EVs-COS (green fluorescence), and then the labeled EVs and EV-COS were co-cultured with rat chondrocytes. After co-culturing for 48 h, the cytoskeleton of chondrocytes was stained with actin red (red fluorescence). It was observed that most chondrocytes displayed intracellular green fluorescence (Fig. 1F), indicating that EVs isolated from rat AMSCs and EVs-COS could be taken up by rat chondrocytes.

### Effects of EVs-COS on cell viability and apoptosis of chondrocytes

**Fig. 2:**
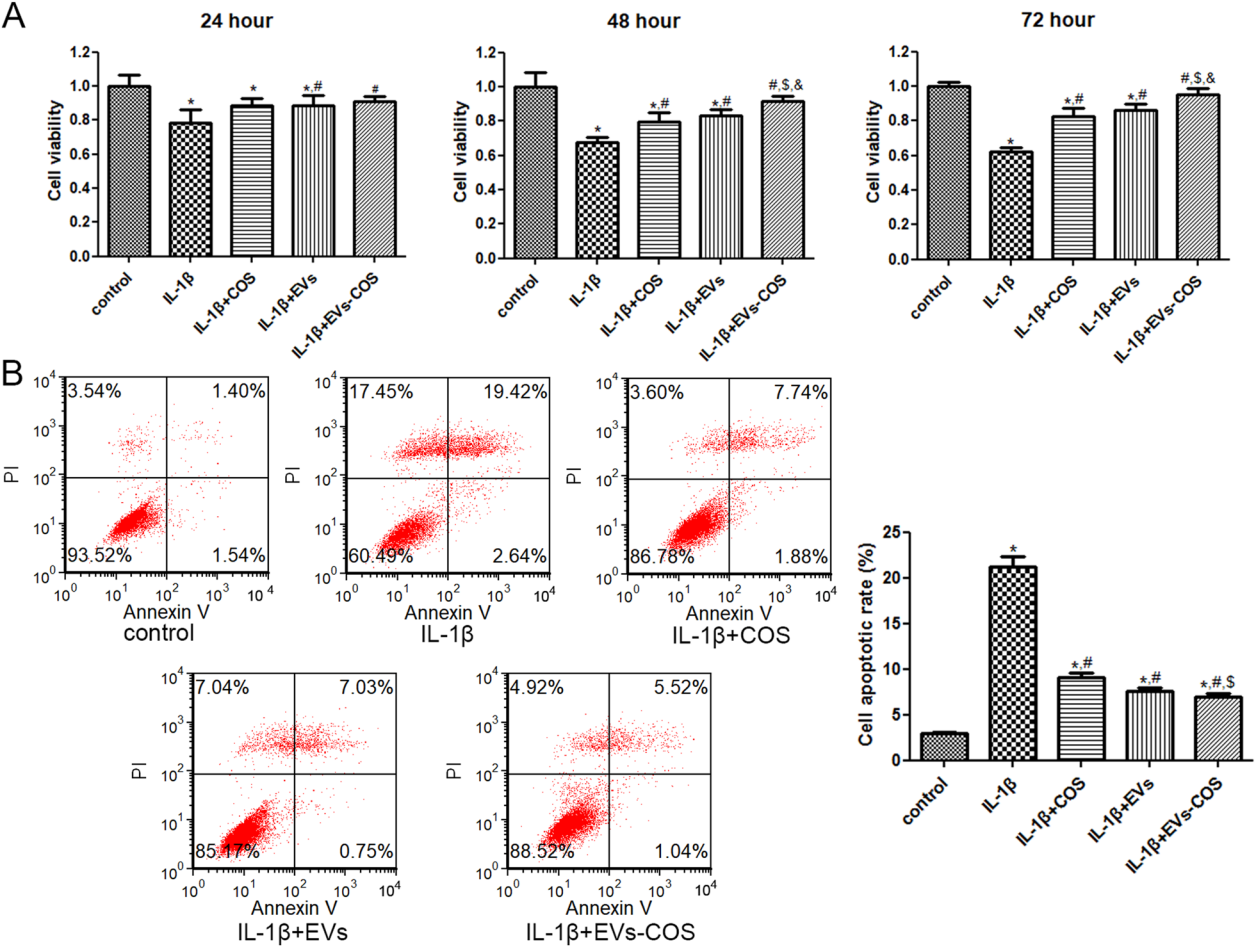
The effects of EVs-COS on the cell viability and apoptosis of chondrocytes induced by IL-1β. **A** After culturing for 24, 48, and 72 h, the cell viability of chondrocytes with different treatments were determined using Cell Counting Kit-8 (CCK-8). **B** The cell apoptosis of chondrocytes with different treatments were detected by flow cytometer. *P < 0.05, compared with the control group; ^#^*P* < 0.05, compared with the IL-1β group; ^$^*P* < 0.05, compared with the IL-1β + COS group; ^&^*P* < 0.05, compared with the IL-1β+EVs group

To understand the effects of EVs-COS on cartilage injury repair, IL-1β was used to establish a chondrocyte injury model. After culturing for 24, 48, and 72 h, cell viability and apoptosis were measured. Cell viability in the IL-1β group was significantly inhibited compared with that in the control group (*P* < 0.05, Fig. 2A). No significant difference in cell viability was found between the IL-1β and IL-1β+COS groups after 24 h (*P* > 0.05). The cell viability in the IL-1β+EVs and IL-1β+EVs-COS groups was increased significantly compared to that in the IL-1β group (*P* < 0.05, Fig. 2A). After being cultured for 48 and 72 h, cell viability in the IL-1β+COS, IL-1β+EVs, and IL-1β+EVs-COS groups was remarkably enhanced compared to that in the IL-1β group (*P* < 0.05). Meanwhile, cell viability in the IL-1β+EVs-COS group was enhanced more significantly than that in the IL-1β+COS group (*P* < 0.05, Fig. 2A). After culturing for 48 h or 72 h, the cell viability in the IL-1β+EVs-COS group was also significantly higher than that in the IL-1β+EVs group (*P* < 0.05, Fig. 2A). Based on these results, chondrocytes treated for 48 h were selected for subsequent experiments.

Flow cytometry was used to determine cell apoptosis in chondrocytes treated with different treatments. The cell apoptotic rates in the control and IL-1β groups were 2.99 ± 0.08% and 21.26 ± 1.11%, respectively, which showed a significant increase in the IL-1β group compared with the control group (*P* < 0.05, Fig. 2B). However, when IL-1β-induced chondrocytes were treated with COS, EVs, and EVs-COS, the apoptotic rates were reduced to 9.12 ± 0.49%, 7.64 ± 0.37%, and 6.94 ± 0.34%, respectively (Fig. 2B). Furthermore, cell apoptosis in the IL-1β+EVs-COS group was significantly lower than that in the IL-1β+COS group (*P* < 0.05, Fig. 2B). These results indicate that IL-1β could significantly suppress the viability of chondrocytes and promote cell apoptosis, while EVs-COS reversed the changes in cell viability and apoptosis induced by IL-1β. In addition, the cell viability and apoptosis changes of EVs-COS were higher than those of COS.

### Effects of EVs-COS on cell migration of chondrocytes

**Fig. 3:**
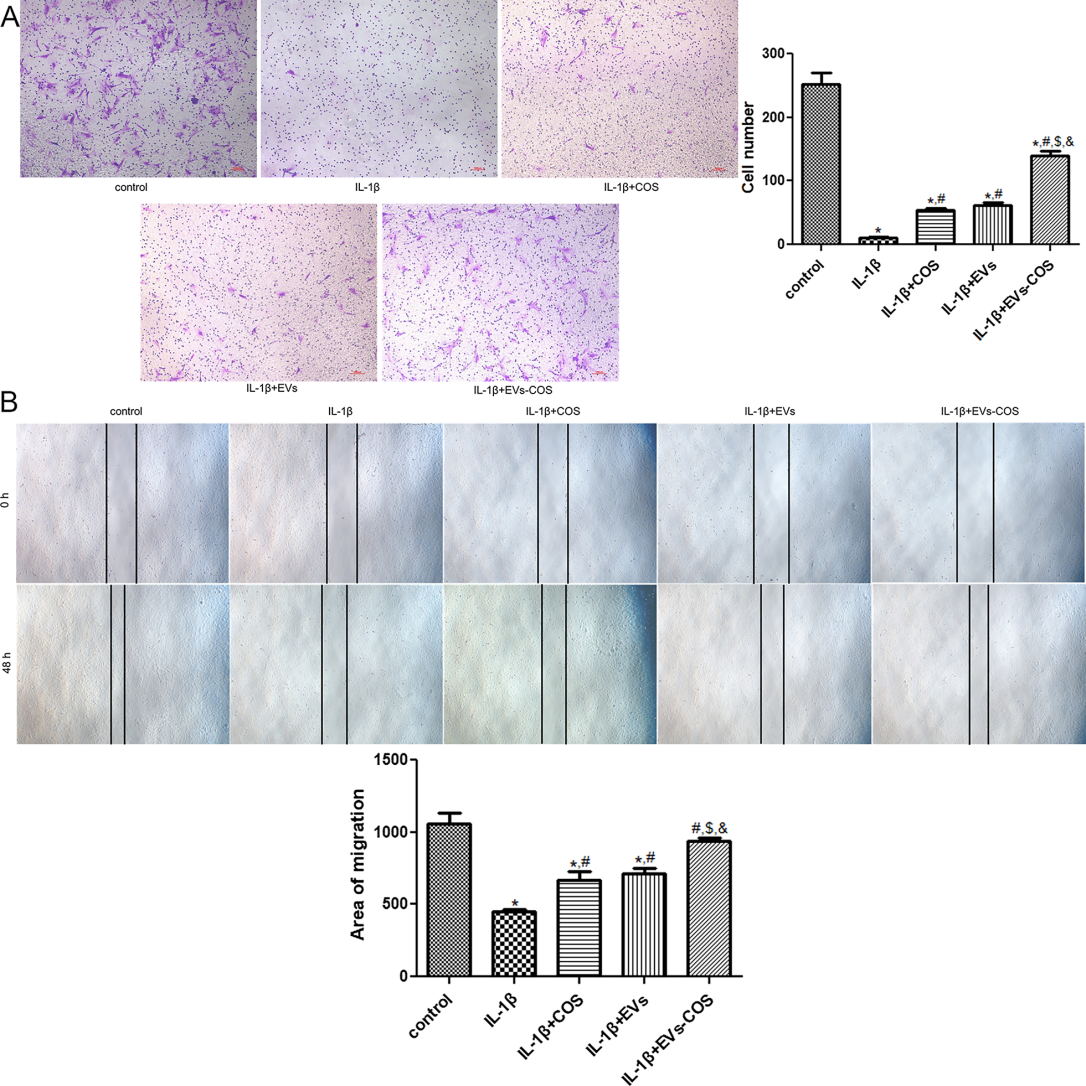
The effects of EVs-COS on the cell migration of chondrocytes induced by IL-1β. **A** The cell number in different groups determined by Transwell. **B** The area of cell migration in different groups measured by scratch test. *P < 0.05, compared with the control group; ^#^*P* < 0.05, compared with the IL-1β group; ^$^*P* < 0.05, compared with the IL-1β+COS group; ^&^*P* < 0.05, compared with the IL-1β+EVs group

The role of EVs-COS in the migration of IL-1β-induced chondrocytes was further investigated using Transwell and scratch tests. Transwell results showed that IL-1β significantly decreased the cell number compared with the control group (*P* < 0.05), whereas COS, EVs, and EVs-COS evidently increased the reduction in cell number induced by IL-1β (*P* < 0.05). The cell number in the IL-1β+EVs-COS group was significantly higher than that in the IL-1β+COS group (*P* < 0.05, Fig. 3A). The results of the scratch test were similar to those of the Transwell test (Fig. 3B). Both Transwell and scratch tests indicated that IL-1β could inhibit chondrocyte migration, while EVs-COS promoted the low migration of chondrocytes caused by IL-1β.

### RT-qPCR and western blot analyses

**Fig. 4:**
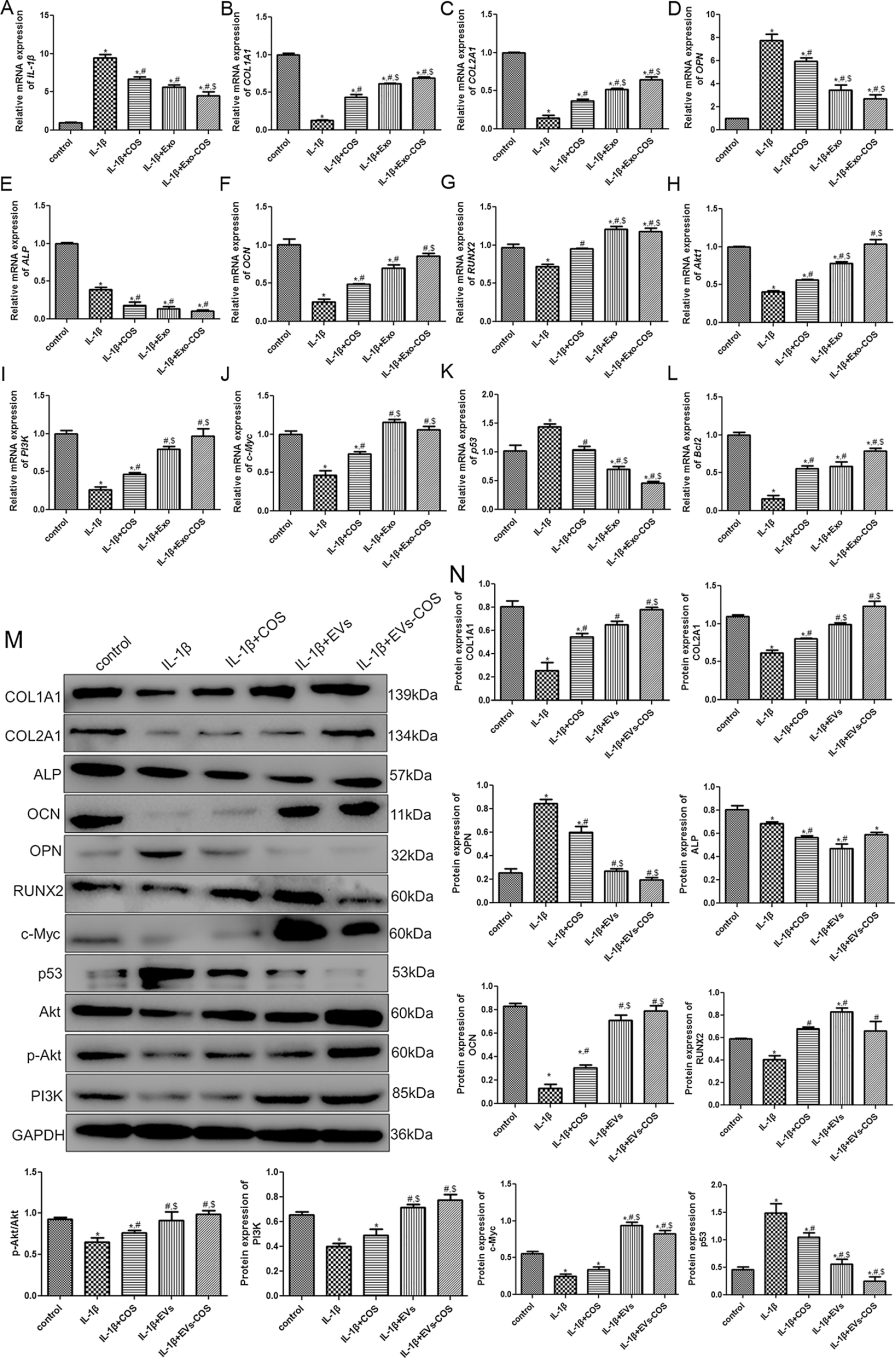
The effects of EVs-COS on the expression levels of related genes and proteins in the chondrocytes with different treatments. The mRNA expression of *IL-1β* (**A**), *COL1A1* (**B**), *COL2A1* (**C**), *OPN* (**D**), *ALP* (**E**), *OCN* (**F**), *RUNX2* (**G**), *Akt1* (**H**), *PI3K* (**I**), *c-Myc* (**J**), *p53* (**K**), and *Bcl2* (**L**). **M** The protein bands determined by Western blot. (**N**) The protein expressions of COL1A1, COL2A1, ALP, OCN, OPN, RUNX2, p-Akt/Akt, PI3K, c-Myc, and p53. *P < 0.05, compared with the control group; ^#^*P* < 0.05, compared with the IL-1β group; ^$^*P* < 0.05, compared with the IL-1β+COS group; ^&^*P* < 0.05, compared with the IL-1β+EVs group

To explore the underlying molecular mechanisms, the expression levels of IL-1β, COL1A1, COL2A1, OPN, ALP, OCN, RUNX2, c-Myc, p53, Bcl2, Akt1, and PI3K were determined using RT-qPCR and Western blotting. Compared with the control group, the mRNA expression of *IL-1β* was significantly upregulated after IL-1β induction (*P* < 0.05), whereas it was significantly downregulated after COS, EVs, and EVs-COS treatment compared with the IL-1β group (*P* < 0.05). Furthermore, the actions of EVs-COS were higher than that of COS (*P* < 0.05, Fig. 4A). The mRNA expression of *COL1A1* and *COL2A1* was evidently downregulated in the IL-1β group compared with the control group (*P* < 0.05), while they were observably upregulated in the IL-1β+COS, IL-1β+EVs, IL-1β+EVs-COS groups compared with the IL-1β group (*P* < 0.05). In addition, the mRNA levels in the IL-1β+EVs and IL-1β+EVs-COS groups were both significantly higher than those in the COS group (*P* < 0.05, Fig. 4B, C). The trend of *OPN* mRNA expression was similar to that of *IL-1β* mRNA expression (Fig. 4D). Interestingly, the mRNA expression of *ALP* was significantly downregulated in the IL-1β group compared to that in the control group (*P* < 0.05) and continued to decline after COS, EVs, and EVs-COS treatments (*P* < 0.05, Fig. 4E). The mRNA expression of *OCN* and *RUNX2* was markedly decreased in the IL-1β group compared to that in the control group (*P* < 0.05), while COS, EVs, and EVs-COS properly restored their expression (Fig. 4F, G). The mRNA expression of Akt1 and PI3K was similar to that of OCN mRNA expression (Fig. 4H, I). Furthermore, compared with the control group, the mRNA expression of *c-Myc* was significantly downregulated after IL-1β induction (*P* < 0.05), while it was notably increased in the COS, EVs, and EVs-COS groups (*P* < 0.05, Fig. 4J). Nevertheless, the trend of *p53* mRNA expression was opposite to that of *c-Myc* expression (Fig. 4K). For *Bcl2*, the tendency of its mRNA expression was in line with that of *c-Myc* mRNA expression (Fig. 4L). Additionally, the results of the Western blot analyses of COL1A1, COL2A1, ALP, OPN, OCN, RUNX2, c-Myc, p53, and PI3K protein expression levels were similar to those of the RT-qPCR (Fig. 4 M, N). The level of p-Akt/Akt in the IL-1β group was significantly downregulated compared to that in the control group (*P* < 0.05), while after treatment with COS, EVs, and EVs-COS, its level was significantly upregulated (*P* < 0.05), and the effect of EVs-COS was better than that of COS (*P* < 0.05, Fig. 4M, N).

### HE staining analysis and immunohistochemical staining of collagen I and collagen II

**Fig. 5:**
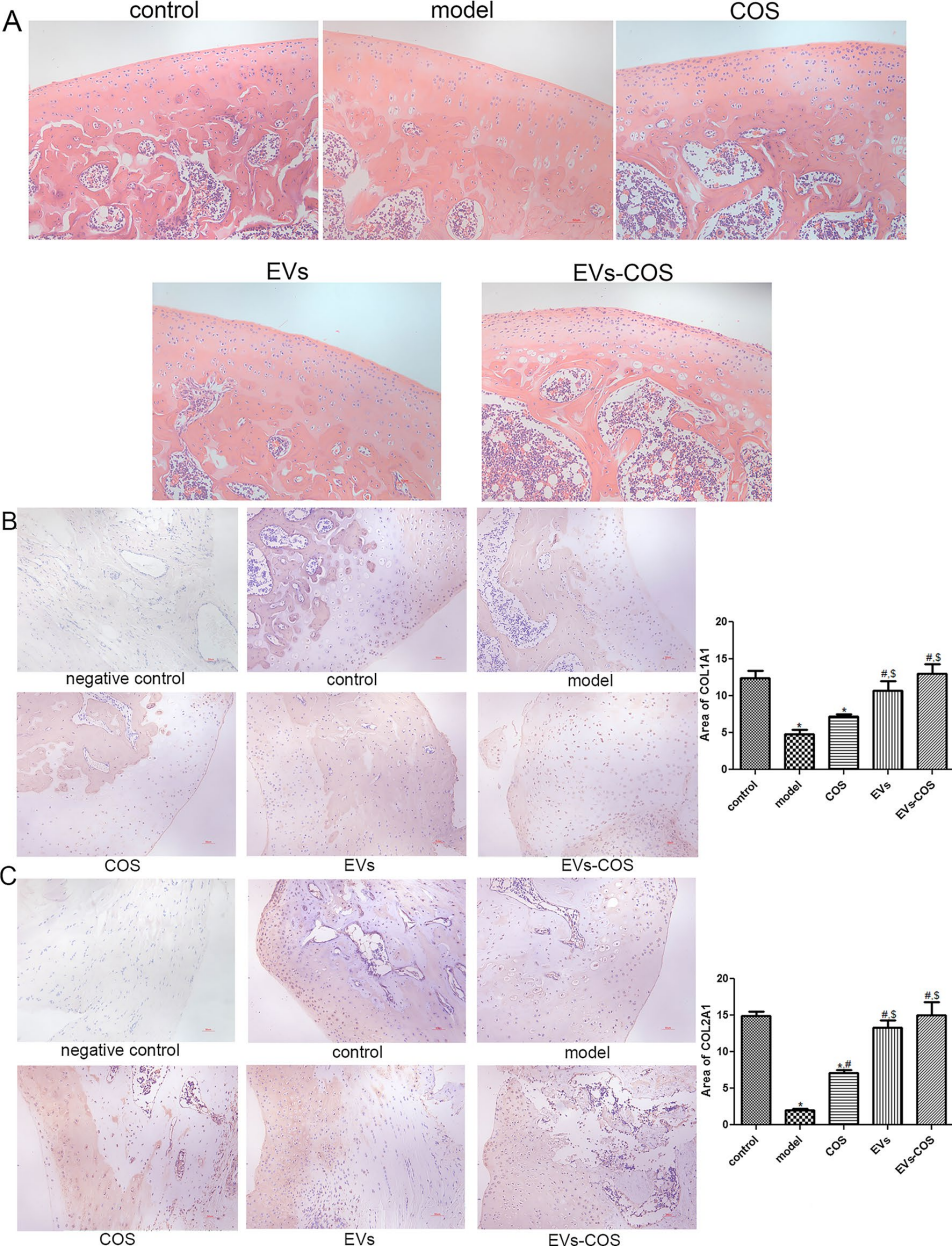
Hematoxylin–eosin (HE) staining analysis and the expressions of collagen I and II in different groups determined by immunohistochemical staining. **A** The representative images of HE staining in different groups. The expression of collagen I (**B**) and II (**C**) in the different groups. *P < 0.05, compared with the control group; ^#^*P* < 0.05, compared with the model group; ^$^*P* < 0.05, compared with the COS group

Cartilage tissues isolated from the rats in the control, injury model, COS, EVs, and EVs-COS groups, and histological changes associated with cartilage injury and EVs-COS treatment in rats were investigated by HE staining of cartilage tissues. The control group showed normal cartilage tissue without infiltration of inflammatory cytokines (Fig. 5A). After modeling, the cartilage tissues in the model group were damaged and swollen, the cartilage matrix was degenerated, and inflammatory cytokines were infiltrated. Cartilage tissue injury was alleviated in the COS, EVs, and EVs-COS groups, and EVs-COS had a better treatment effect on cartilage tissue injury (Fig. 5A).

Subsequently, the expression of collagen I and II was determined using immunohistochemical staining. As shown in Fig. 5B, the expression of COL1A1 in the model and COS groups was significantly lower than that in the control (*P* < 0.05). EVs and EVs-COS significantly increased the expression of COL1A1 compared to the model group (*P* < 0.05). Meanwhile, COL2A1 expression was dramatically downregulated in the model group compared to that in the control group (*P* < 0.05), while COS, EVs, and EVs-COS markedly upregulated its expression (*P* < 0.05). No significant difference was observed among the control, EV, and EVs-COS groups (*P* > 0.05, Fig. 5C). These results indicated that the expression of COL1A1 and COL2A1 could be downregulated after cartilage injury, and that EVs-COS could reverse the trends (i.e., expression reduction) caused by cartilage injury.

### Screening of DEGs in the same expression pattern and functional analyses

After comparison, a total of 2,091, 503, and 412 DEGs were identified between the COS and model groups, between EVs-COS and model groups, as well as between EVs-COS and COS groups, respectively (Additional file 3: Figure S3). Thereafter, by comparing the DEGs between COS vs. model, EVs-COS vs. model, and EVs-COS vs. COS, and retaining DEGs in at least two comparison groups, 760 DEGs were obtained for further expression trend clustering analysis (Additional file 4: Table S1).

**Fig. 6:**
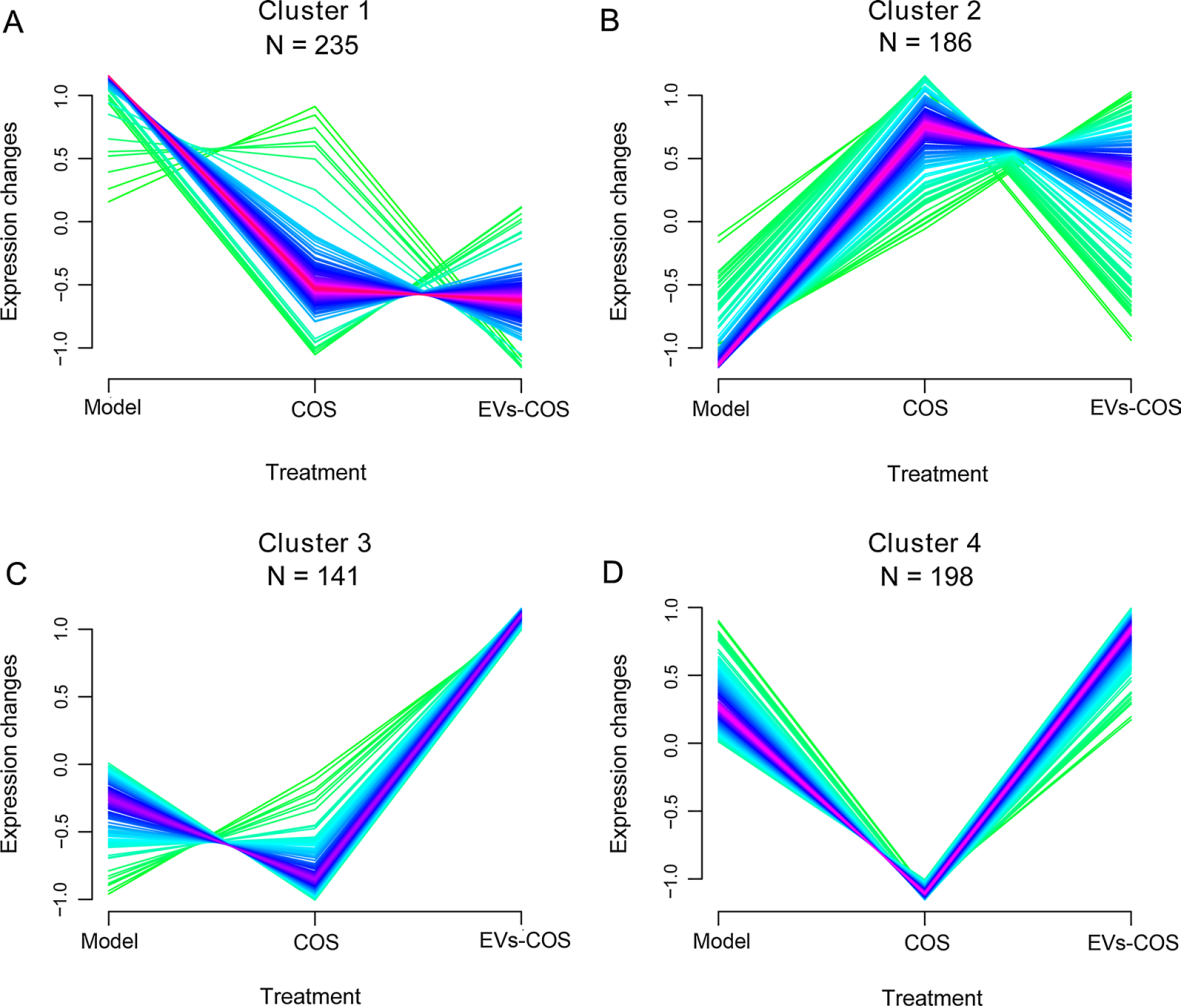
Clustering diagram of expression patterns of the obtained differentially expressed genes (DEGs) based on Mfuzz algorithm

Afterwards, the Mfuzz algorithm was used to analyze the expression patterns of the obtained 760 DEGs, and the expression trend of the target genes under different treatments was observed. Four expression patterns, clusters 1, 2, 3, and 4, were clustered (Fig. 6), and the DEGs in each are shown in Additional file 4: Table S1.

**Fig. 7:**
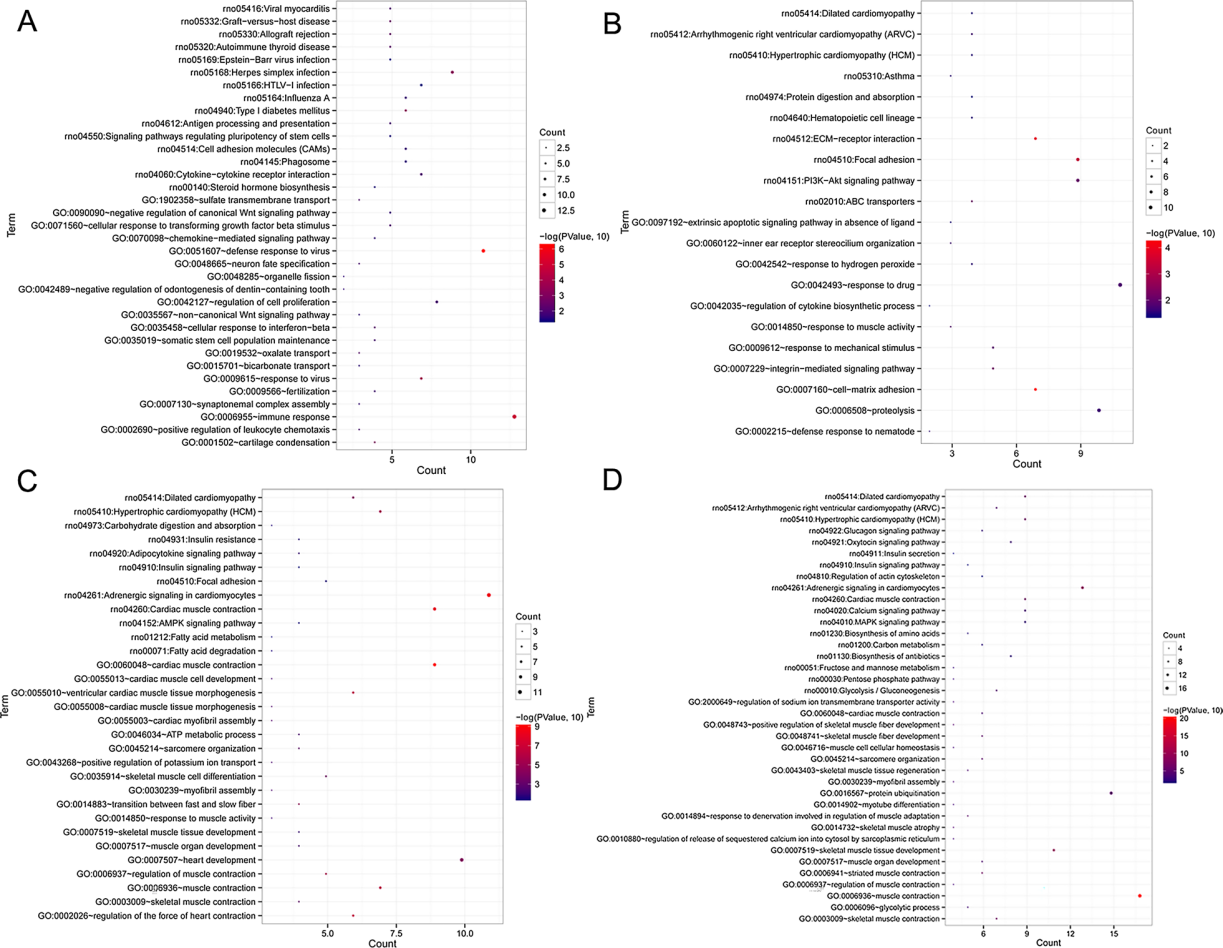
Functional analyses of the DEGs in the clusters 1 (**A**), 2 (**B**), 3 (**C**) and 4 (**D**)

In addition, the DEGs in each cluster were used for the BP and KEGG enrichment analyses (Additional file 5: Table S2). It was found that the DEGs in cluster 1 were significantly enriched in allograft rejection, signaling pathways regulating pluripotency of stem cells, phagosomes, negative regulation of canonical Wnt signaling pathway (*SFRP5*, *SOX9*, and *GLI1*), cellular response to transforming growth factor beta stimulus, regulation of cell proliferation, and cartilage condensation (Fig. 7A). The DEGs in cluster 2 were strongly associated with ECM-receptor interaction, focal adhesion, PI3K-Akt signaling pathway (*COL1A1*, *COL3A1*, *ITGA4*, *BAD,* and *FOXO3*), and proteolysis (Fig. 7B). Meanwhile, the DEGs in cluster 3 were involved in adrenergic signaling in cardiomyocytes, AMPK signaling pathway (*PPP2R3A*, *CPT1B*, and *ACACB*), and positive regulation of potassium ion transport (Fig. 7C). Lastly, the DEGs in cluster 4 played important roles in the regulation of actin cytoskeleton, MAPK signaling pathway (*CACNA1S*, *CACNG1*, and *MAPK12*), pentose phosphate pathway, sarcomere organization, muscle contraction, skeletal muscle tissue development, and myofibril assembly (Fig. 7D).

### Validation of sequencing results by RT-qPCR

**Fig. 8:**
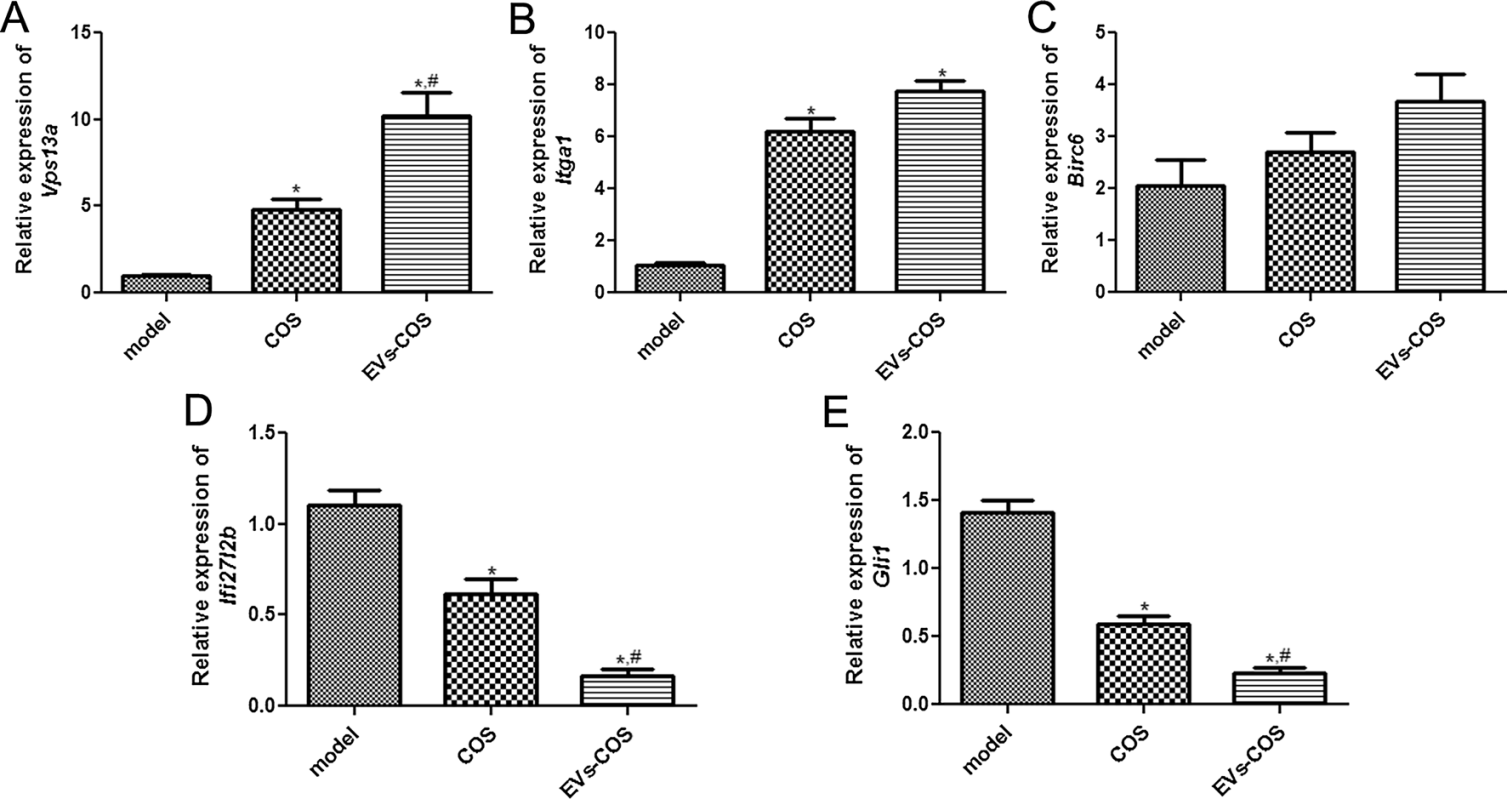
Validation of DEGs by real-time quantification PCR. The mRNA expression levels of *Vps13a* (**A**), *Itga1* **(B**), *Birc6* (**C**), *Ifi27l2b* (**D**), and *Gli1* (**E**). *P < 0.05, compared with the model group; ^#^*P* < 0.05, compared with the COS group

Five DEGs, including three upregulated and two downregulated in the COS and EVs-COS groups, were chosen for the validation of sequencing results by using RT-qPCR. It is clear that the expression levels of *Vps13a* and *Itga1* were significantly upregulated in the COS and EVs-COS groups compared with the model group (*P* < 0.05, Fig. 8A, B). Furthermore, the expression of *Ifi27l2b* and *Gli1* in the COS and EVs-COS groups were obviously lower than those in the model group (*P* < 0.05, Fig. 8D, E). These results indicated that the tendency of *Vps13a*, *Itga1*, *Ifi27l2b,* and *Gli1* expression determined by RT-qPCR were consistent with those analyzed by sequencing. However, there was no significant difference in *Birc6* expression among the model, COS, and EVs-COS groups (*P* > 0.05, Fig. 8C). All these results implied that the consistency rate of sequencing findings and RT-qPCR results was 80%, which represents a relatively high reliability.

## Discussion

OA is the injury of articular cartilage caused by multiple factors, such as mechanical trauma or inflammation, which seriously affects people’s health and quality of life [5]. EVs are carriers of many bioactive substances and play important roles in cell-to-cell communication [29]. Chitin and its derivative chitosan have been reported as bone repair materials that promote osteoblast growth and healing [22]. In this study, COS was packaged into rat AMSC-derived EVs, and the effects of EVs-COS on cartilage injury repair both in vitro and in vivo were explored. Previous studies have suggested that MSC-derived EVs can attenuate OA [30], and that COS can improve cartilage damage, thus, preventing and treating OA [31]. Our research found that EVs-COS could enhance the viability and migration of chondrocytes induced by IL-1β and suppress their apoptosis. A previous study demonstrated that human MSC-derived EVs can relieve IL-1β-induced chondrocyte viability repression and apoptosis induction, thus alleviating chondrocyte damage and OA [32]. Another study has shown that hyaluronic acid/chitosan nanoparticles can deliver curcuminoids to increase cell proliferation while suppressing apoptosis and inflammatory response in the chondrocytes of knee OA [33]. Taken together, we speculated that EVs-COS may promote cartilage injury repair and ameliorate OA by promoting the viability and migration of chondrocytes and inhibiting cell apoptosis.

To investigate the mechanisms of the treatment of OA by EVs-COS in vitro, RT-qPCR and Western blotting were performed to measure the expression levels of related genes and proteins. After cartilage damage, IL-1β, OPN, and p53 were significantly upregulated, while COL1A1, COL2A1, OCN, RUNX2, p-Akt/Akt, PI3K, c-Myc, and Bcl2 were markedly downregulated. Meanwhile, COS and EVs-COS could reverse the expression induced by cartilage injury, and the actions of EVs-COS were stronger. IL-1β, a pro-inflammatory cytokine, has been used to establish an inflammatory model of chondrocytes in vitro [34]. COL1A1 and COL2A1, encoding type I and II collagen, are chondrocyte-specific markers that are essential for linear growth, bone development, and the structural framework of cartilage [35]. A study by Jain et al. reported that IL-1β pre-treatment inhibited COL1A1 and COL2A1 expression in chondrocytes, while lyophilized platelet-rich plasma treatment increased the level of COL1A1 [36]. OCN, OPN, and RUNX2 are osteoblast-related genes, and their expression is closely related to the maturation and differentiation of osteoblasts [37]. RUNX2 has been reported to be a crucial gene in osteogenesis that induces the expression of OPN and OCN [37]. A previous study showed that lipopolysaccharide (LPS) significantly reduced osteoblastic differentiation in MC3T3-E1 cells by downregulating RUNX2, OCN, and OPN expression [38], while resveratrol promoted LPS-induced MC3T3-E1 cell differentiation and increased the expression of RUNX2, OCN, and OPN. P53, a well-known apoptotic gene, is involved in the cell cycle, DNA repair, cell apoptosis, and senescence [39]. c-Myc is a primary regulator of cell proliferation and transformation, and its abnormal expression is the basis of many cancers [40]. Bcl2, an anti-apoptotic factor, has been reported to participate in apoptosis and the growth and differentiation of chondrocytes [41]. Mei et al. indicated that PRMT5 knockdown could inhibit the growth and migration of endometrioid adenocarcinoma cells and promote cell apoptosis by downregulating c-Myc and Bcl2, and upregulating p53 [42]. Akt is the main effector of PI3K signal transduction and modulates a variety of pathways, such as the inhibition of cell apoptosis, stimulation of cell growth, and regulation of cell metabolism [43]. Bakuchiol promotes chondrocyte proliferation and injured cartilage remodeling through the PI3K/Akt and ERK1/2 pathways [44]. These findings are consistent with our experimental results, and suggest that EVs-COS could affect viability, apoptosis, and migration of chondrocytes by regulating chondrocyte-specific genes (COL1A1 and COL2A1), osteoblast-related genes (OCN, OPN, and RUNX2), apoptosis-related genes (c-Myc, p53, and Bcl2), and the Akt/PI3K pathway, thereby improving cartilage injury repair and OA.

Subsequently, an in vivo cartilage injury model was constructed, and chondrocytes were isolated from different groups. The expression of COL1A1 and COL2A1 was determined using immunohistochemical staining, and the results of COL1A1 and COL2A1 in vivo were consistent with those in vitro. Afterwards, the chondrocytes in the model, COS, and EVs-COS groups were used for sequencing, and a total of 760 DEGs were identified, which were clustered into four expression patterns (clusters 1, 2, 3, and 4). The DEGs in all clusters were associated with negative regulation of the canonical Wnt, PI3K-Akt, AMPK, and MAPK signaling pathways. The canonical Wnt signaling pathway, mediated by β-catenin, has been reported to promote the differentiation of osteoblasts, bone formation, and bone resorption [45]. An experimental OA mouse model was established, which revealed that the inhibition of the Wnt/β-catenin signaling pathway improved the severity of OA and reduced the degeneration of cartilage and synovitis in vivo [46]. Our in vitro experiments have shown that EVs-COS can affect cartilage injury repair through the PI3K-Akt signaling pathway, a finding confirmed by the in vivo results. It has been reported that the AMPK signaling pathway plays important roles in regulating cellular energy balance, resisting oxidative stress, and alleviating inflammation [47]. A previous study reported that LPS induced the activation of the AMPK/NF-κB pathway and higher levels of chondrocyte apoptosis, while miR-137 overexpression inhibited the AMPK/NF-κB pathway and reversed OA development [48]. Additionally, the activation of the MAPK signaling pathway can contribute to the expression of pro-inflammatory cytokines and chemokines in chondrocytes of OA, while blockage of this pathway can inhibit chondrocyte apoptosis and prevent the recruitment of inflammatory cells that may lead to the degradation of cartilage [49]. These findings confirm that EVs-COS can promote cartilage injury repair and relieve OA through negative regulation of the canonical Wnt, PI3K-Akt, AMPK, and MAPK signaling pathways.

Through sequencing, we also found that *SFRP5*, *SOX9,* and *GLI1* were significantly enriched in the negative regulation of the canonical Wnt signaling pathway; *COL1A1*, *COL3A1*, *ITGA4*, *BAD*, and *FOXO3* were significantly enriched in the PI3K-Akt signaling pathway; *PPP2R3A*, *CPT1B,* and *ACACB* were significantly enriched in the AMPK signaling pathway; and *CACNA1S*, *CACNG1*, and *MAPK12* were significantly enriched in the MAPK signaling pathway. A previous study has shown that the level of *SFRP5* is closely related to bone density and bone formation [50]. *SOX9*, a key transcription factor in chondrocytes, has been widely reported and studied for cartilage development [51]. *GLI1*, *COL1A1*, *COL3A1,* and *FOXO3* have also been reported to be related to cartilage injury repair and the occurrence and progression of OA [52–54]. Therefore, the hypothesis that EV-COS may promote cartilage injury repair by regulating *SFRP5*, *SOX9*, *GLI1*, *COL1A1*, *COL3A1,* and *FOXO3* may be correct*.*

However, few reports have shown the roles of *ITGA4*, *BAD*, *PPP2R3A*, *CPT1B*, *ACACB*, *CACNA1S*, *CACNG1*, and *MAPK12* in cartilage injury repair. *ITGA4* encodes a member of the integrin α chain family, and its high level is associated with many diseases, such as chronic lymphocytic leukemia [55], gastrointestinal stromal tumors [56] and colorectal cancer [57]. *BAD* plays a key role in mitochondria-dependent apoptosis, and its inactivation contributes to the development of rheumatoid arthritis by conferring apoptosis resistance in synovial sub lining macrophages [58]. *PPP2R3A*, a major serine/threonine phosphatase, is involved in a variety of cellular processes, including cell apoptosis, proliferation, DNA repair, and autophagy [59], and its knockdown inhibits the proliferation, migration, and invasion of hepatoma carcinoma cells [60]. *CPT1B* and *ACACB* are genes related to fatty acid oxidation [61] and are associated with obesity and diabetes. *CACNA1S* and *CACNG1*, calcium channel related genes, could serve as novel therapeutic targets for remodeling the extracellular matrix of aging lamina propria [62]. *MAPK12*, which may be a risk factor for the prognosis of bladder cancer patients [63], encodes p38γ, which is mainly expressed in the skeletal muscle. Combining these studies with our results, it can be inferred that *ITGA4*, *BAD*, *PPP2R3A*, *CPT1B*, *ACACB*, *CACNA1S*, *CACNG1*, and *MAPK12* may play important roles in promoting cartilage injury repair by EVs-COS. However, the specific effects of these genes on OA should be further verified both in vitro and in vivo.

## Conclusions

EVs-COS could facilitate cartilage injury repair and have better protective effects on OA by promoting the viability and migration of chondrocytes, suppressing cell apoptosis, and regulating COL1A1, COL2A1, OCN, OPN, RUNX2, c-Myc, p53, Bcl2, and the Akt/PI3K pathway. Sequencing revealed that negative regulation of the canonical Wnt, PI3K-Akt, AMPK, and MAPK signaling pathways may be strongly associated with cartilage injury repair. *SFRP5*, *SOX9*, *GLI1*, *COL1A1*, *COL3A1*, *FOXO3*, *GA4*, *BAD*, *PPP2R3A*, *CPT1B*, *ACACB*, *CACNA1S*, *CACNG1*, and *MAPK12* may be potential targets for EV-COS-promoting cartilage injury repair. Our findings have revealed the underlying pathogenesis of OA and laid the foundation for the therapy of cartilage injuries and damage-based OA with EVs-COS as a novel potential drug.

## Methods

### Isolation and identification of EVs

Rat AMSCs were purchased from Cyagen Biosciences Inc. (Guangzhou, China). Rat AMSCs were cultured in α-minimum essential medium (α-MEM; Thermo Fisher Scientific, Waltham, MA, USA), supplemented with 10% fetal bovine serum (FBS, Thermo Fisher Scientific) and 1% penicillin/streptomycin (Thermo Fisher Scientific), and maintained in an incubator with 5% carbon dioxide at 37 °C.

The extraction methods for EVs from rat AMSCs have been described previously [64]. Briefly, rat AMSCs were cultured in medium with serum but without EVs for 48 h, and the cell supernatants of rat AMSCs were collected. The obtained cell supernatants were transferred to a new 50 mL tube, and centrifuged at 500×*g* for 4 min at 4 °C. The supernatant was then transferred to a new tube and centrifuged at 2000×*g* for 30 min at 4 °C. The supernatant was then mixed with the same amount of pre-cooled polyethylene glycol (PEG, 16%) and incubated at 4 °C overnight. After centrifugation at 10,000×*g* for 60 min at 4 °C, the supernatant was discarded, and 1 mL phosphate buffer saline (PBS) was added to the sediment. After blending, the mixture was centrifuged at 100,000×*g* for 70 min at 4 °C. The sediment was resuspended in 200 μL PBS, and the EVs were stored at − 80 °C for subsequent experiments.

The concentrations of EVs were measured using a BCA assay kit (Thermo Fisher Scientific), according to the manufacturer’s instructions. Subsequently, a Nanosight NS300 particle size analyzer (NTA; Malvern Panalytical, Malvern, UK) was used to determine the particle distribution of EVs following the method described by Soares Martins et al. [29]. The morphology of isolated EVs was visualized by transmission electron microscopy (TEM, JEOL LTD, Peabody, MA, USA) according to a previous study [65]. Furthermore, the protein expression of CD63, TSG101, CD9, and calnexin were detected in the EVs and cells via Western blotting with their corresponding antibodies (1:2000) [66].

### Binding of EVs with COS

The concentration of EVs was adjusted to 40 μg/mL using Dulbecco’s modified Eagle’s medium (DMEM, Thermo Fisher Scientific), and 640 μg/mL COS (Tokyo Chemical Industry, TCI, Tokyo, Japan) was prepared with DMEM. Thereafter, EVs were mixed with COS in equal volumes, and the mixture was incubated with shaking at 37 °C for 1 h. After centrifugation at 120,000×*g* for 60 min, the sediment was resuspended in 200 μL PBS, and EVs-COS conjugates were obtained. A TEM was then used to determine the morphology of the EVs-COS, and NTA was used to measure the size distribution of the EVs-COS.

### Isolation and culture of rat chondrocytes

Rat chondrocytes were isolated from the cartilage tissues of SPF Wistar rats weighing 180 ± 20 g, as described previously [34]. Briefly, the rats were killed by cervical dislocation, and cartilage tissues from the knee were collected under sterile conditions. After washing thrice with PBS, the cartilage tissues were cut into small pieces (1 mm^3^), and then 0.2% type II collagenase solution (Beyotime Biotechnology, Shanghai, China): 0.25% trypsin solution (Beyotime Biotechnology; v/v, 4:1; including DNase) was added. After digestion at 37 °C for 6 h, DMEM/F12 medium (Thermo Fisher Scientific) was added to terminate the digestion. After filtering, the collected chondrocyte suspension was centrifuged at 1000 rpm for 5 min, and the chondrocytes were cultured in DMEM/F12 medium supplemented with 10% FBS and 1% penicillin/streptomycin. Afterwards, the chondrocytes were inoculated into a 25 cm^2^-cell culture plate and passaged when the cell confluence reached 80–90%.

### Immunohistochemistry staining

The chondrocytes were characterized by immunohistochemical staining for type II collagen. The chondrocytes were washed with PBS and fixed with 4% paraformaldehyde for 20 min. After washing thrice with PBS, 0.5% Triton X-100 (Sigma-Aldrich, USA) was added, and the mixture was incubated at 22 ± 3 °C for 15 min. After washing, 3% H_2_O_2_ was added, and the resulting mixture was incubated at 22 ± 3 °C for 15 min. The cells were then washed and then incubated with anti-collagen II antibody (1:1000, Proteintech, Chicago, USA) or anti-collagen I antibody (1:1000, NOVUS) overnight. Afterwards, the cells were washed and then treated with horseradish peroxidase (HRP)-labeled secondary antibody (Goat anti-rabbit IgG, Jackson ImmunoResearch Laboratories, Inc.) for 1 h. After washing, the cells were incubated with diaminobenzidine (DAB, Beyotime Biotechnology) for 5 min, and the cells were then redyed with hematoxylin (Sigma-Aldrich). The color of the cells was observed under a microscope at 200× magnification.

### Co-culture of EVs or EVs-COS and chondrocytes

EVs and EVs-COS were labeled with PKH67 (green fluorescence) using a PKH67 staining kit (MINI67-1KT, Sigma-Aldrich) following the manufacturer’s protocols. Briefly, Diluent C (900 μL) was added to the EVs (100 μL) or EVs-COS (100 μL), and then PKH67 (4 μL) was added. After incubation at 22 ± 3 °C for 5 min, 2 mL of 1% BSA was added to bind excess dye. After centrifugation at 120,000×*g* for 60 min at 4 °C, the sediment was resuspended in 300 μL PBS, and PKH67-labeled EVs and EVs-COS were successfully prepared.

Afterwards, the chondrocytes were seeded in a 24-well plate at a density of 3 × 10^4^ cells/well and cultured in serum-free medium overnight. The next day, 20 μL PKH67-labeled EVs and EVs-COS were added to the cells. After co-culturing for 48 h, the cells were washed with PBS and fixed with 4% paraformaldehyde for 20 min. After washing, the cells were treated with 0.1% Triton X-100 for 5 min. Afterwards, the cells were washed, added with 1% BSA, and incubated at 22 ± 3 °C for 30 min. After removing the 1% BSA, the cells were stained with actin red (red fluorescence) in the dark at 22 ± 3 °C for 20 min. Thereafter, the cells were stained with DAPI (Thermo Fisher Scientific), and images were taken using a laser scanning confocal microscope (TCS SP8, Leica Microsystems, Inc., USA).

### Cell viability and cell apoptosis assays

The chondrocytes were seeded into 96-well plates and divided into five groups: control, IL-1β, IL-1β+COS, IL-1β+ EVs, and IL-1β+EVs-COS groups. The cells in the IL-1β, IL-1β+COS, IL-1β+EVs, and IL-1β+EVs-COS groups were treated with 10 ng/mL IL-1β for 24 h first, and then with PBS, 320 μg/mL COS, 20 μg/mL EVs, and EVs-COS (20 μg/mL EVs + 320 μg/mL COS), each, for another 24 h, 48 h, and 72 h. Cells in the control group were not treated.

The cell viability of the chondrocytes was examined using a Cell Counting Kit-8 (CCK-8, Beyotime Biotechnology) according to the manufacturer’s recommendations. Briefly, 10 mL of CCK-8 reagent was added to each well and incubated for 2 h. The absorbance at 450 nm was determined using a microplate reader (Multiskan MK3; Thermo Fisher Scientific).

After culturing for 48 h, the cell suspension was harvested and used for the cell apoptosis assay using the Annexin V-FITC/PI apoptosis assay kit (Beyotime Biotechnology). The harvested cell suspension was centrifuged at 1000 rpm for 5 min and resuspended in 1 × binding buffer (100 μL). Afterwards, 5 μL each of Annexin V-FITC and propidium iodide (PI) were added to the cells. After incubation at 22 ± 3 °C in the dark for 15 min, 400 μL of 1 × binding buffer was added to the mixture. Images of apoptosis were acquired using a flow cytometer.

### Cell migration assay

Transwell and scratch tests were performed to evaluate the cell migration. For Transwell, chondrocytes with different treatments were collected and resuspended in 0.1% BSA medium. Afterwards, 200 μL of cell suspension was added to the upper chamber of the Transwell chambers (pore size 8 μm; Guangzhou Jet Bio-Filtration Co., Ltd., Guangzhou, China), while complete medium was added to the lower chamber. After culturing for another 24 h, the chambers were removed, and the supernatant was removed. The cells were then washed and then fixed with 4% paraformaldehyde for 10 min. After washing with PBS, the cells were stained with crystal violet (Beyotime Biotechnology) at 22 ± 3 °C for 20 min. After washing the excess dye and drying, the cells were observed and photographed under an inverted microscope.

For the scratch test, the chondrocytes were seeded in a 6-well plate at a density of 5 × 10^5^ cells/well, and the cells were subjected to different treatments. The cell layer was then scratched with a micropipette, and the cell medium was removed. After washing with PBS thrice, serum-free medium was added. Images of the cells were captured at 0 h and 48 h.

### Real-time quantitative PCR (RT-qPCR)

Total RNA was extracted from the chondrocytes with different treatments using RNAiso Plus (TRIzol, TAKARA, Japan) according to the manufacturer’s instructions. The concentrations and quality of the total RNA were measured using a microplate reader. Next, PrimeScript™ RT Master Mix (TAKARA) was used to reverse transcribe the total RNA into cDNA following the manufacturer’s instructions. The RT-qPCR reaction was initiated at 95 °C for 2 min, followed by 40 cycles of 95 °C for 15 s, 60 °C for 60 s, 95 °C for 15 s, 60 °C for 60 s, and 95 °C for 15 s. *GAPDH* was used as a housekeeping gene, and the sequences of all primers are shown in Table 1. Relative mRNA expression levels were calculated using the 2^−ΔΔCt^ method [67].

**Table 1:** The sequences of all primers

Primer	Sequence (5′–3′)
GAPDH-ratF	AGACAGCCGCATCTTCTTGT
GAPDH-ratR	CTTGCCGTGGGTAGAGTCAT
IL-1β-rF	CCCTGCAGCTGGAGAGTGTGG
IL-1β-rR	TGTGCTCTGCTTGAGAGGTGCT
COL1A1-rF	GCATCAGGGTTTCAGAGCA
COL1A1-rR	CGTTGGGTCATTTCCACAT
COL2A1-rF	TTCCTCCGTCTACTGTCCACTGA
COL2A1-rR	CTACATCATTGGAGCCCTGGAT
OPN-rF	CCAGCCAAGGACCAACTACA
OPN-rR	AGTGTTTGCTGTAATGCGCC
ALP-rF	GCAGTATGAATTGAATCGGAACAAC
ALP-rR	ATGGCCTGGTCCATCTCCAC
c-Myc-rF	CTGCTGTCCTCCGAGTCCTC
c-Myc-rR	GGGGGTTGCCTCTTTTCCAC
OCN-rF	ACCATCTTTCTGCTCACTCTGCT
OCN-rR	CCTTATTGCCCTCCTGCTTG
RUNX2-rF	CCACAGAGCTATTAAAGTGA
RUNX2-rR	AACAAACTAGGTTTAGAGTCATCAAGC
Akt1-rF	CTTTATTGGCTACAAGGAACGG
Akt1-rR	CAGTCTGAATGGCGGTGGT
PI3K-rF	ACGGCAATGTGGAGCAGA
PI3K-rR	GTCGTAGCCAATCAGGGAG
Bcl2-rF	GATTGTGGCCTTCTTTGAGTTC
Bcl2-rR	CAGATGCCGGTTCAGGTACT
p53-rF	GCTGAGTATCTGGACGACAGG
p53-rR	AGCGTGATGATGGTAAGGATG
Vps13a-rF	GCATTGAGAATACCGTTTG
Vps13a-rR	GAATCCCATCTGCTTTGT
Birc6-rF	CACTCTGGGTCTACTGCT
Birc6-rR	ATTAGGGTGACATGAAATAC
Itga1-rF	CATCCCTCATAACACCACC
Itga1-rR	GTTCCCATCCTCCATCTT
Ifi2712b-rF	GGAGGAGTTGTGGCTGTG
Ifi2712b-rR	AAATCTTGCATTGGAGGC
Gli1-rF	CTCCCTCGTGGCTTTCAT
Gli1-rR	GCGGCAGTCCGTCTCATA

### Western blot

RIPA lysis buffer (Beyotime Biotechnology) was used to isolate total protein from the cells with different treatments, and the protein concentrations were determined using a BCA assay kit (Thermo Fisher Scientific). Protein samples (20 μg) were separated by 10% SDS-PAGE, transferred to polyvinylidene fluoride membranes, and blocked with 5% skim milk at 37 °C for 2 h. After washing thrice with PBST (PBS with 1% Tween-20), the membranes were incubated with primary antibodies against c-Myc (1:1000, CST), ALP (1:500, Abcam), OCN (1:1000, NOVUS), RUNX2 (1:1000, Abcam), OPN (1:600, Abcam), Akt (1:1000, CST), p-Akt (1:2000, CST), PI3K (1:2000, Proteintech), COL1A1 (1:1000, NOVUS), COL2A1 (1:1000, Proteintech), p53 (1:1000, Proteintech), and GAPDH (1:1000, Proteintech) at 4 °C overnight. After washing, the membranes were then incubated with the secondary antibody (1: 1000, Jackson ImmunoResearch Laboratories, Inc.) at 37 °C for 2 h. After washing, the protein bands were visualized using ECL system.

### Animal experiments in vivo

Thirty SPF female Wistar rats weighing 180 ± 20 g were purchased from Shanghai SLAC Laboratory Animal Co., Ltd. (Shanghai, China). All rats were maintained at 22–25 °C and 20–25% humidity conditions, with a 12 h light/dark cycle. During the experiments, all rats had ad libitum access to water and food. After acclimatization for seven days, the rats were randomly and equally divided into five groups (n = 6 for each group): control, model, COS, EVs, and EVs-COS. Except for the rats in the control group, the other rats were used to construct a cartilage injury model, as described previously [68]. Briefly, the rats were anesthetized by intraperitoneal injection of 3% pentobarbital sodium (0.1 mL/100 g), and then the skin at the knee joint was incised. The joint capsule was cut from the medial edge of the patella, and the articular cartilage on the patellofemoral joint surface was exposed. Subsequently, a full-thickness cartilage defect model with a diameter of 1 mm and a depth of 0.2 mm was prepared at 2 mm above the intercondylar fossa. After modeling, the articular cavity was rinsed, and the joint capsule and skin incisions were sutured. After the operation, penicillin (30,000 U/time) was injected intramuscularly once a day for three consecutive days. Two weeks later, the rats in the COS, EVs, and EVs-COS groups were injected with COS (2 mg), EVs (100 μg), and EVs-COS (100 μg), respectively, in the articular cavity once a week for 8 weeks. Rats in the control group were not treated.

After the experiments, cartilage tissues were obtained from the different groups. A portion of the tissues was used for hematoxylin–eosin (HE) staining and type I and type II collagen immunohistochemistry staining [69], while the rest of the model, the COS and EVs-COS groups, were sent to Yanzai Biotechnology (Shanghai) Co., Ltd (China) for RNA sequencing. In addition, five genes were chosen for RT-qPCR to further verify the reliability of the sequencing data. All animal experiments were conducted in accordance with the National Medical Advisory Committee (NMAC) guidelines and approved by the Ethics Committee of China Medical University (CMU2021187).

### Analysis of sequencing data

The samples were divided into three comparison groups: COS vs. model, EVs-COS vs. model, and EVs-COS vs. COS. Differentially expressed genes (DEGs) between COS and model, EVs-COS and model, as well as EVs-COS and COS were screened using Limma package version 3.34.0 in R3.6.1 (https://bioconductor.org/packages/release/bioc/html/limma.html) [70]. The threshold values for DEG selection were false discovery rate (FDR) < 0.05 and |log_2_ fold change (FC)|> 1. After that, Mfuzz version 2.42.0 (http://www.bioconductor.org/packages/release/bioc/html/Mfuzz.html) [71] in R3.6.1 was used to analyze the expression patterns of the obtained DEGs, and the gene clustering of expression modules was obtained. Finally, the DEGs in each cluster were submitted for biological process (BP) of gene ontology (GO) and Kyoto Encyclopedia of Genes and Genomes (KEGG) pathway enrichment analyses using DAVID version 6.8 (https://david.ncifcrf.gov/) [72, 73]. Statistical significance was set at *P* < 0.05.

### Statistical analysis

All experiments were performed at least thrice, and data are expressed as the mean ± standard deviation (SD). GraphPad Prism 5 (San Diego, CA, USA) was used for the statistical analyses. For multiple comparisons, a one-way analysis of variance (ANOVA) with a post-hoc Tukey test was applied. Differences were considered statistically significant at P < 0.05.

## Supplementary Information

**Additional file 1: Figure S1.** Screen of the optimal concentrations of extracellular vesicles (EVs) and chitosan oligosaccharide (COS). **A** The cell viability of chondrocytes with different concentrations of EVs. **P < 0.01, compared with 0 μg/mL EVs. **B** The cell viability of chondrocytes with different concentrations of COS. *: *P* < 0.05, compared with 0 μg/mL COS; **P < 0.05, compared with 0 μg/mL COS.

**Additional file 2: Figure S2.** The identification of rat chondrocytes extracted from rats’ cartilage tissues by type II collagen immunohistochemical staining.

**Additional file 3: Figure S3.** The differentially expressed genes (DEGs) between COS and model groups, between EVs-COS and model groups, as well as between EVs-COS and COS groups.

**Additional file 4: Table S1.** The differentially expressed genes (DEGs) in at least two comparison groups.

**Additional file 5: Table S2.** DEGs in each cluster used for biological process and Kyoto Encyclopedia of Genes and Genomes (KEGG) enrichment analyses.

## Data Availability

The data that support the findings of this study are available from the corresponding author upon reasonable request.

## Abbreviations

COS: Chitosan oligosaccharides
EVs: Extracellular vesicles
AMSCs: Adipose mesenchymal stem cells
OA: Osteoarthritis
EVs-COS: EVs-COS conjugates
TEM: Transmission electron microscopy
NTA: Nanosight NS300 particle size analyzer
PI: Propidium iodide
NMAC: National Medical Advisory Committee
IL-1β: Interleukin-1β
COL1A1: Collagen type I alpha 1 chain
COL2A1: Collagen type II alpha 1 chain
OPN: Secreted phosphoprotein 1
ALP: Alkaline phosphatase
OCN: Osteocalcin
RUNX2: RUNX family transcription factor 2
c-Myc: Transcriptional regulator Myc-like
p53: Tumor protein p53
Bcl2: BCL2 apoptosis regulator
Akt1: AKT serine/threonine kinase 1
PI3K: Phosphatidylinositol 3-kinase, putative
SFRP5: Secreted frizzled related protein 5
SOX9: SRY-box transcription factor 9
GLI1: LI family zinc finger 1
COL3A1: Collagen type III alpha 1 chain
ITGA4: Integrin subunit alpha 4
BAD: BCL2 associated agonist of cell death
FOXO3: Forkhead box O3
PPP2R3A: Protein phosphatase 2 regulatory subunit B'' alpha
CPT1B: Carnitine palmitoyltransferase 1B
ACACB: Acetyl-CoA carboxylase beta
CACNA1S: Calcium voltage-gated channel subunit alpha1 S
CACNG1: Calcium voltage-gated channel auxiliary subunit gamma 1
MAPK12: Mitogen-activated protein kinase 12
