# Supplementary figures and images for: Chitosan oligosaccharides packaged into rat adipose mesenchymal stem cells-derived extracellular vesicles facilitating cartilage injury repair and alleviating osteoarthritis
Source: J Nanobiotechnology. 2021 Oct 26;19:343. doi: 10.1186/s12951-021-01086-x (PMC8549296; doi:10.1186/s12951-021-01086-x)

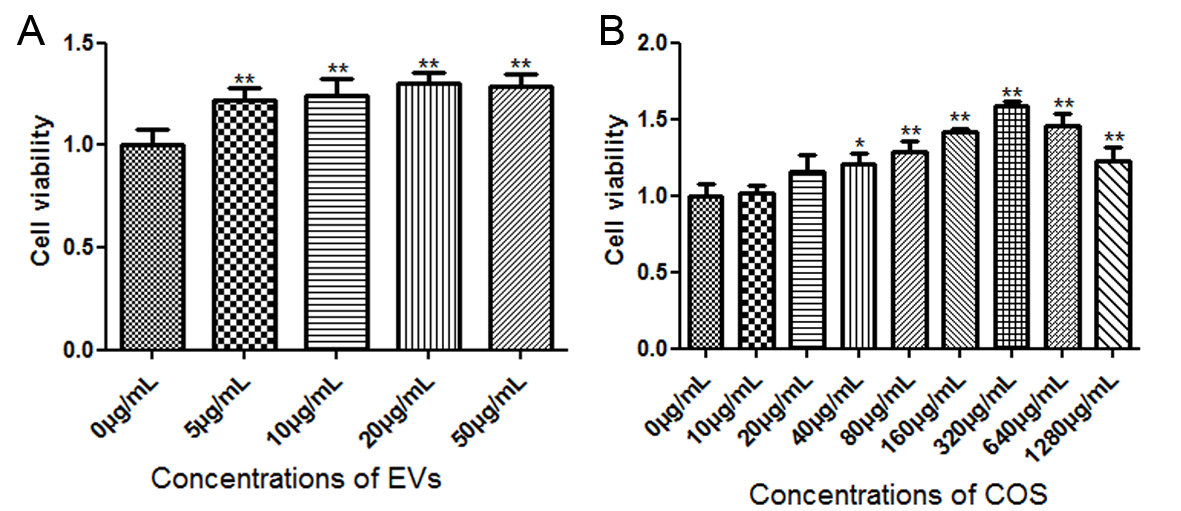

Supplement: Supplementary file 1 — Additional file 1: Figure S1. Screen of the optimal concentrations of extracellular vesicles (EVs) and chitosan oligosaccharide (COS). A The cell viability of chondrocytes with different concentrations of EVs. **P < 0.01, compared with 0 μg/mL EVs. B The cell viability of chondrocytes with different concentrations of COS. *: P < 0.05, compared with 0 μg/mL COS; **P < 0.05, compared with 0 μg/mL COS. [file 12951_2021_1086_MOESM1_ESM.tif]

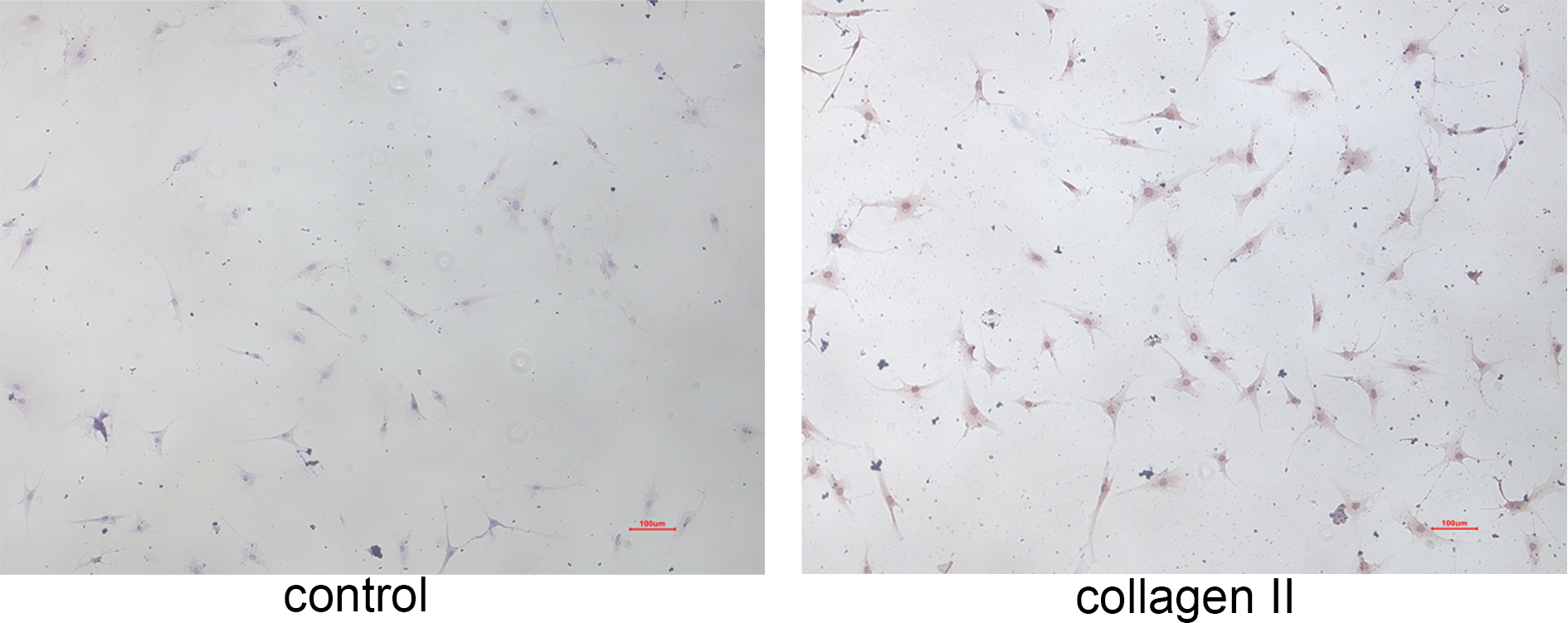

Supplement: Supplementary file 2 — Additional file 2: Figure S2. The identification of rat chondrocytes extracted from rats’ cartilage tissues by type II collagen immunohistochemical staining. [file 12951_2021_1086_MOESM2_ESM.tif]

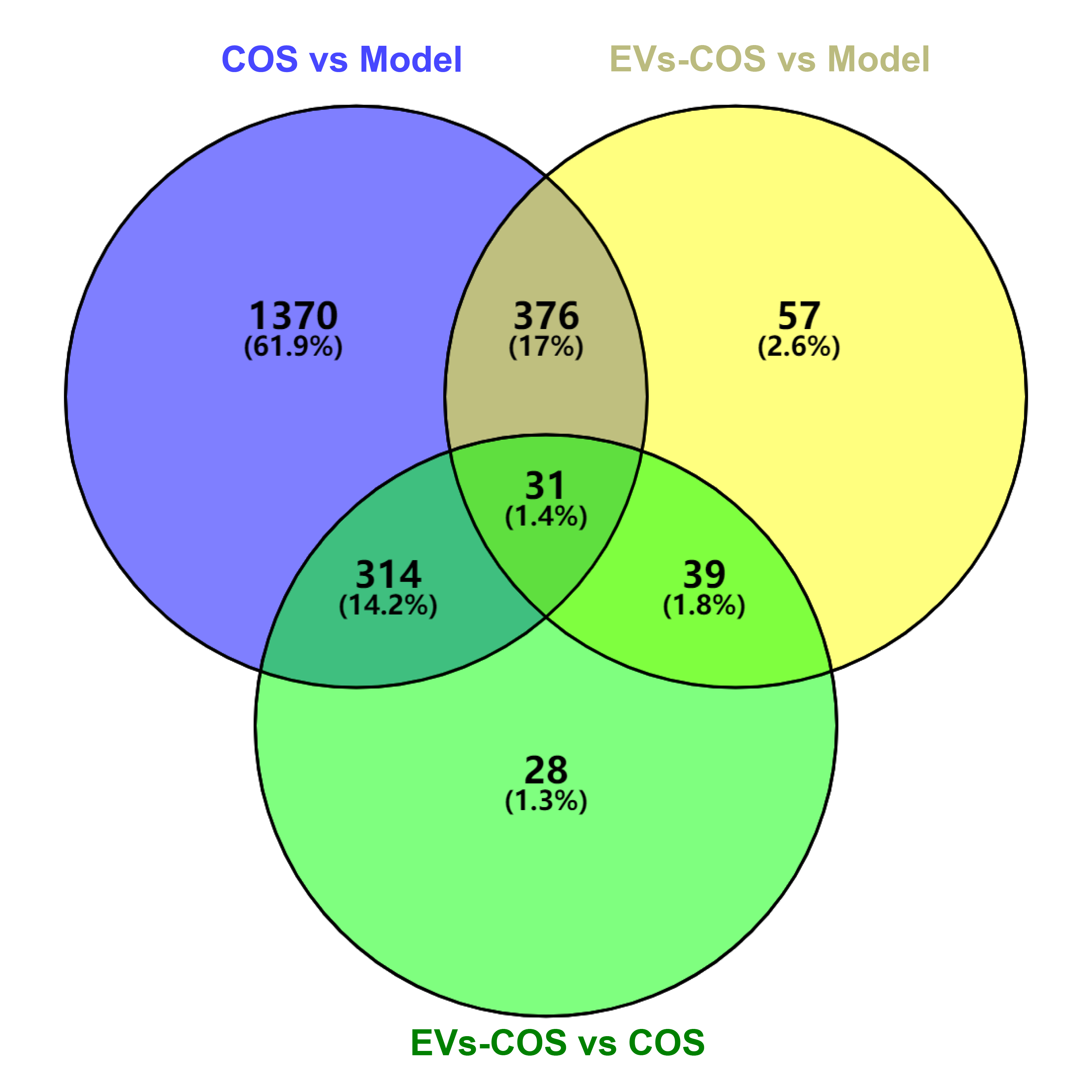

Supplement: Supplementary file 3 — Additional file 3: Figure S3. The differentially expressed genes (DEGs) between COS and model groups, between EVs-COS and model groups, as well as between EVs-COS and COS groups. [file 12951_2021_1086_MOESM3_ESM.tif]
